# Editorial: iSensor and iMedicine for human health

**DOI:** 10.3389/fchem.2022.1107145

**Published:** 2022-12-01

**Authors:** Chengchao Chu, Yan Zhang

**Affiliations:** ^1^ School of Medicine, Xiamen University, Xiamen, China; ^2^ School of Chemistry and Chemical Engineering, University of Jinan, Jinan, China; ^3^ Key Laboratory of Optic-Electric Sensing and Analytical Chemistry for Life Science, MOE, Qingdao University of Science and Technology, Qingdao, China

**Keywords:** sensor, wearable devices, electrochemistry, electroluminescence, *in vitro*, *in vivo*

Up to now, precise diagnosis and treatment of diseases have always been the hot Research Topic in the fields of analytical chemistry, biology and medicine (Nie et al., 2021; Hou et al., 2022). In order to serve public health and obtain a better understanding of diseases, in-depth researches have been carried out, with much more attention attached to the occurrence, mechanism, prevention and diagnosis of diseases, as well as safe and effective treatments, which play a critical role in disease events. Thus, iSensor (intelligentized sensor) and iMedicine (intelligentized medicine) were proposed to satisfy the high requirements for the recent medical environment.

Diagnosis of disease can be divided into *in vitro* diagnosis and *in vivo* diagnosis. *In vivo* diagnosis can be further divided into two methods, wearable diagnosis (Zhu et al.) and *in vivo* imaging, both of which are already involved in important clinical practice. In addition, *in vitro* diagnosis refers to the diagnosis of diseases through the analysis of blood, urine, saliva, tears and even breath gas collected in the body, and combined with the analysis of active ingredients, and thus understand the disease by sensors. Among various *in vitro* diagnostic strategy, electrochemical immunosensor has received more and more attention due to its convenient and rapid detection. To increase the sensitivity of electrochemical sensor, methylene blue (MB) modified MWCNT (MWCNT-MB) was coated on the surface of glassy carbon electrode (GCE) to increase the electron transfer property (Zhang et al.). Then, polydopamine was synthesized on MWCNT to connect the anti- carcinoembryonic antigen (CEA) antibody (Ab). With the addition of CEA, the redox signal of MWCNT-MB decreased due to the reduction of electron transfer efficiency. Thus, the immunosensor was applied to the detection of CEA with a low limit of detection (LOD). Similarly, chitosan-reduced graphene oxide composite and gold nanoparticles were modified on the surface of GCE to increase the electrochemical signal (Chang et al.). After the modification of Ab, bone gamma-carboxyglutamate protein was immobilized on GCE, reducing the electrochemical signal of added electrochemical probe ([Fe(CN)_6_]^3−^/^4−^).

Furthermore, electroluminescence (ECL) was another research hotspot for its low background, fast detection speed and high detection sensitivity (Fereja et al., 2020; Liu et al., 2022). In this Research Topic, the vertically ordered mesoporous silica-nanochannel film (VMSF) was coated on the surface of ITO electrode, in which positively charged Ru(bpy)_3_^2+^enriched inner the nanochannel (Ma et al.). In a further step, prostate-specific antigen (PSA) Ab was modified on the surface of VMSF/ITO electrode. In addition, the PSA could specifically bind with Ab and thus resisting the physical absorption of Ru(bpy)_3_^2+^ into the nanochannel, resulting in the decrease of ECL signal. Finally, the constructed ECL sensor was applied to the detection of PSA, and the immunosensor possessed a low LOD. Meanwhile, Wei et al. coated polyethylene terephthalate (PET) on ITO electrode, and further modified with VMSF. The clindamycin was confirmed to enhance the ECL of Ru(bpy)_3_^2+^, and the VMSF/PET-ITO sensor could detect clindamycin, using Ru(bpy)_3_^2+^ as ECL luminophores. In a further study, Gong et al. constructed a three-dimensional (3D) ECL platform using VMSF modified macroporous 3D graphene electrode. Unlike traditional electrode, 3D graphene showed high diffusion/mass transfer efficiency, benefiting for the ECL detection. The Ru(bpy)_3_^2+^/tri-n-propylamine (TPrA) was applied to the detection of 4-chlorophenol using the ECL sensor, in which the ECL signal of Ru(bpy)_3_^2+^/TPrA was quenched by 4-chlorophenol. Moreover, chlorpheniramine could promote the ECL signal of Ru(bpy)_3_^2+^, and the chlorpheniramine could be detected using the ECL sensor, with a LOD of 430 nM. Therefore, the proposed VMSF modification is an effective strategy to increase the sensitivity of ECL sensors.

Other than the traditional single-mode sensor, dual-mode or multi-mode sensor could improve detection rate and reduce the background influence (Zhang et al. 2020). For example, Tan et al. constructed a colorimetric/fluorescent dual-mode sensor using Co_3_O_4_ nanozymes, which was then applied in the detection of H_2_O_2_ and glucose with high sensitivity; Wan et al. constructed a colorimetric/fluorescent dual-mode sensor using nitrogen-doped graphene quantum dot and applied in the detection of H_2_O_2_, ascorbic acid and acid phosphatase with high sensitivity. In addition, researchers modified multi-biosensor on one chip for simultaneous detection of multi-biomarker, enabling fast quantification of the multi-biomarker associated disease. (Meng et al. 2022)

In recent years, the intelligent method has also been applied in drug development. Cheng et al. synthesized a non-alcoholic steatohepatitis treated compound YWS01125. To evaluate the pharmacokinetics of YWS01125, an ultraperformance liquid chromatography-tandem mass spectrometry (UPLC-MS/MS) strategy was applied. The pharmacokinetics studies indicated that YWS01125 could be an advanced drug to treat with non-alcoholic steatohepatitis. Furthermore, with the continuous development of nano/micro-medicine, the use of nano/micro-materials for drug delivery or nano-therapy arose (Shi et al.; Wang et al.). All in all, it could be concluded that the future of iSensor and iMedicine depends on three key factors: 1) new types of *in vitro* and *in vivo* diagnostic equipment; 2) advanced diagnostic probes, imaging probes, and smart medicines; 3) effective data integration and analysis.
